# Common breastfeeding problems experienced by lactating mothers during the first six months in Kinshasa

**DOI:** 10.1371/journal.pone.0275477

**Published:** 2022-10-12

**Authors:** Pélagie Babakazo, Marc Bosonkie, Eric Mafuta, Nono Mvuama, Mala-Ali Mapatano

**Affiliations:** 1 Kinshasa School of Public Health, University of Kinshasa, Kinshasa, Democratic Republic of the Congo; 2 Centre Mère et Enfant Barumbu, Kinshasa, Democratic Republic of the Congo; University of Washington, UNITED STATES

## Abstract

**Introduction:**

Breastfeeding has numerous advantages for infant, mother and society. However, many mothers discontinue breastfeeding due to problems they encounter. This study aimed to identify problems commonly experienced by breastfeeding mothers during the first six months in Kinshasa.

**Methods:**

A prospective cohort study was carried out in Kinshasa from October 2012 to July 2013. A total of 422 mother-infant couples were recruited shortly after being discharged from twelve maternity facilities in Kinshasa and followed-up for six months. Interviews were conducted at the mother’s house during the first week after birth, and thereafter at monthly intervals for six months. Data included mother’s sociodemographic characteristics, the breastfeeding problems she experienced and information on child’s feeding. Incidences of breastfeeding problems encountered during different periods were calculated as well as their confidence intervals.

**Results:**

Cracked or sore nipples, insufficient production of milk and breast engorgement were the most commonly experienced problems by lactating mothers. The problems occurred mainly during the first week (17.1%; CI_95%_ 13.7–21.1) and the rest of the first month (16.2%; CI_95%_ 12.8–20.3).

**Conclusions:**

The first month after birth presents the most risk for the occurrence of breastfeeding problems. Mothers should be supported as soon as possible after delivery, to improve their breastfeeding performance and to be informed on how to maintain breast milk supply.

## 1. Introduction

Breastfeeding is the optimal way of feeding for neonates and infants. Furthermore, its duration is of vital importance for the child’s development and health. The World Health Organization (WHO) recommends that infants be breastfed exclusively for the first six months of life and then, when other foods have been introduced, continue to be breastfed until aged two years or beyond [1]. "Exclusive breastfeeding" is defined as feeding the baby with only breast milk, without any other food or drink, not even water [2]. "Predominant breastfeeding" on the other hand means that the infant’s main source of nourishment is breast milk, while child’s diet contains other liquids such as water, water-based drinks or fruit juice [2].

Breastfeeding provides short-term and long-term health and economic advantages to children, women, and society. Those benefits go far beyond nutrition. Children who are breastfed are less at risk of morbidity and mortality, compared to those who are not breastfed [3]. Moreover, they are protected against infections and malocclusion, they enjoy a high intelligence quotient, and they may have reduced long-term risk of becoming obese and diabetic [4]. For lactating women, breastfeeding ensures spacing of births and prevents breast and ovarian cancers [4]. Scaling up breastfeeding to a near universal level could prevent 823 000 child deaths and 20 000 maternal deaths per annum [5]. Thus, it contributes to achieving the first and second targets of the third Sustainable Development Goal, related respectively to maternal and child mortalities. Furthermore, not breastfeeding is associated with an economic loss of about $302 billion annually or 0.49% of world gross national income [5].

Despite these advantages, rates of exclusive breastfeeding in most countries remain below the global recommendation. During 2012, the World Health Assembly set a target for 2025 to increase exclusive breastfeeding rates in the first six months up to at least 50% [6]. However, in low-income and middle-income countries, only 37% of children younger than six months of age are exclusively breastfed [4].

In the Democratic Republic of the Congo (DRC), almost all infants are breastfed and just over half of children less than six months old are exclusively breastfed [7]. Although the country is moving towards the 2025 WHO target, from 37% in 2010 [8] to 48% in 2014 [9], and 52% in 2018 [7], one could hypothesize that the trend would be faster if some obstacles were overcome. According to the WHO, countries that are already at or near 50% exclusive breastfeeding have to continue to strive for improvements because of the health and economic benefits of exclusive breastfeeding [6]. Moreover, despite the improvement noticed during the last decade, the mortality rate among children under five in the DRC remains far higher from 25 deaths per 1000 live births, the second target of the third Sustainable Development Goal [10]. This rate moved from 158 deaths per 1000 live births in 2010 [8] to 104 deaths per 1000 live births in 2014 [6] and 70 deaths per 1000 live births in 2018 [7].

Exclusive breastfeeding is often hindered by a number of problems. A systematic review on obstacles to exclusive breastfeeding in low- and middle-income countries show that breastfeeding problems are commonly reported [11]. Studies in Pakistan, Tanzania and Ghana have reported that breast and nipple problems, including swollen and sore breasts, breast abscesses, mastitis, and cracked or sore nipples, are important barriers to EBF [12–14]. While most women initiate breastfeeding, many discontinue due to problems encountered rather than maternal choice. Breastfeeding mothers need to be informed about strategies to prevent problems; at the same time, they need support in order to overcome problems they may face. Health care providers have been identified by mothers as the primary source of information on breastfeeding [15, 16]. Thus, the former could support mothers in preventing breastfeeding problems or managing them if they occurred. This is all the more convincing in a country like the DRC where rates of breastfeeding initiation as well as antenatal visits and delivery in a health care facility are high [7].

Therefore, health care providers should be aware of common problems experienced by breastfeeding mothers and when they are most likely to occur. This study aimed to identify breastfeeding problems commonly experienced by breastfeeding mothers during the first six months in Kinshasa and to determine when the problems are more frequent.

## 2. Methods

### 2.1. Design, setting and participants

We re-analysed data from a prospective cohort study, on infant feeding practices, carried out among mother-infant couples in Kinshasa from October 2012 to July 2013. The study design and enrolment of participants are described elsewhere [17]. Briefly, mother-infant couples were randomly selected from 12 maternity facilities in Kinshasa during the first week after childbirth.

A multiple stage sampling technic was used to recruit the participants. Firstly, two heath districts among the six of Kinshasa were randomly selected. Secondly, in the selected heath districts, twelve maternities were randomly selected using the list of all eligible maternities. To be eligible, at least 60 births should occur in the maternity facility per month. Regarding the mother-infant couples, to be eligible, the mother needed to be at least 18 years old, given birth to a single living full-term child who was free of any serious health conditions that would require transfer to an intensive care unit. All eligible women who gave birth in the selected maternity facility during the study period, were recruited. Each mother-infant couple was followed-up during home visits until the infant was fed with non-human milk or with complementary food, or until six months for nursing mothers who had practiced exclusive or predominant breastfeeding for the duration of that period.

### 2.2. Definition of concepts

The researchers in this study used the WHO definitions [2] of breastfeeding and infant feeding practices, as follows: a child was considered exclusively breast-fed, when he or she had received only breast milk with no other liquids (including water) or solids; predominately breast-fed when he or she had received breast milk as the main source of nourishment but also liquids such as water, water-based drinks or fruit juice; and having been complementary fed when that feeding consisted of any food or liquid, including non-human milk and formula, along with breast milk.

The duration of exclusive or predominant breastfeeding was defined as the infant’s age at the introduction of non-human milk or complementary foods; in other words, the age at which the infant began complementary feeding. This duration was initially measured in days, and then converted to months during data analysis.

### 2.3. Data collection

Six interviewers, female and fluent in French and Lingala, the most widely spoken language in Kinshasa, were trained to collect the data. Face-to-face individual interviews were conducted at the mother’s home at seven times: during the first week after birth, and at 1, 2, 3, 4, 5, and 6 months. During the first visit, sociodemographic data were collected as follows—mother’s age, education level, marital status, main occupation and parity. During this first visit, the mother was also asked about her infant feeding plans. At each visit, the mother was asked whether she had experienced any problem or difficulty with breastfeeding since her previous visit or since the delivery for her first visit. If her answer was in the affirmative, she was asked to describe the breastfeeding problems that she had experienced. More than one breastfeeding problem could be reported. The mother was also asked how her infant was fed the previous day. If non-human milk or complementary food was mentioned, the mother was asked when she had started feeding her infant with the food or milk in question.

### 2.4. Data analysis

Characteristics of study participants were summarized using the median and the interquartile range (IQR) for continuous variables and proportions for categorical variables namely: occupation, level of education, marital status, parity and planned infant feeding. Frequency and type of breastfeeding problems experienced over the six first months were presented in frequency distribution tables.

The Kaplan Meier Method was used to determine the median duration of exclusive and predominant breastfeeding. For these survival analyses, the duration of exclusive or predominant breastfeeding was right censored at the child’s age the moment the mother-infant couple dropped out of the study and at six months for mothers who continued exclusive or predominant breastfeeding up to the end of the study. The child’s death was also considered as a censoring event. All statistical analysis was performed in Stata version 14. Statistical significance was set at p < 0.05.

### 2.5. Ethical considerations

The study was approved by the Ethical Committee of the Kinshasa School of Public Health (ESP/CE/001/2012). During the first visit, all participants were informed about the study and the freedom to leave any time should they feel uncomfortable. They all gave their written informed consent to participate. All research procedures were in accordance with the Declaration of Helsinki.

## 3. Results

### 3.1. Participants’ characteristics

A total of 422 mother-infant couples were enrolled in the study. Their characteristics are shown in Table 1. Almost three mothers out of five (57.2%) were 20 to 29 years old. Their median age was 26 years (IQR 22 to 31). Less than two percent of mothers (1.9%) had never attended school and nearly half (49.1%) had completed primary education. Most of them (85.3%) were married or living with a partner. Regarding their main occupation, nearly half of the participants (48.3%) were housewives. Almost four out of ten participants (38.4%) were first time mothers. Regarding planned infant feeding, nearly nine mothers out of ten (88.1%) planned to breastfeed their babies during their first days of life. Of these mothers, almost four in ten (42.2%) planned to breastfeed exclusively for the first six months.

**Table 1:** Characteristics of study participants.

	Number n = 422	Percent
**Age (years)**		
<20	47	11.2
20–29	241	57.2
≥30	133	31.6
**Education level**		
Never been at school	8	1.9
Primary	207	49.1
Secondary	179	42.4
University	28	6.6
**Marital status**		
Living with a partner	360	85.3
Single	62	14.7
**Occupation**		
Housewife	204	48.3
Small trade	106	25.1
Hairdresser/dressmaker	68	16.1
Paid job	20	4.7
Student	18	4.3
Farm worker	6	1.4
**Parity**		
1	162	38.4
2–3	167	39.6
≥4	93	22.0
**Planned feeding for the baby**		
Breastfeeding	372	88.1
Mixed breastfeeding	43	10.2
No planning	7	1.7
**Planned duration of EBF (n = 372)**		
Less than six months	210	56.5
Six months	157	42.2
More than six months	5	1.3

Among the 422 mother-infant couples enrolled, 405 (96.0%) were followed for the full six months. Attrition was due to the migration of the family (16/17) or to the child’s death (1/17).

### 3.2. Breastfeeding practices

**Fig 1:**
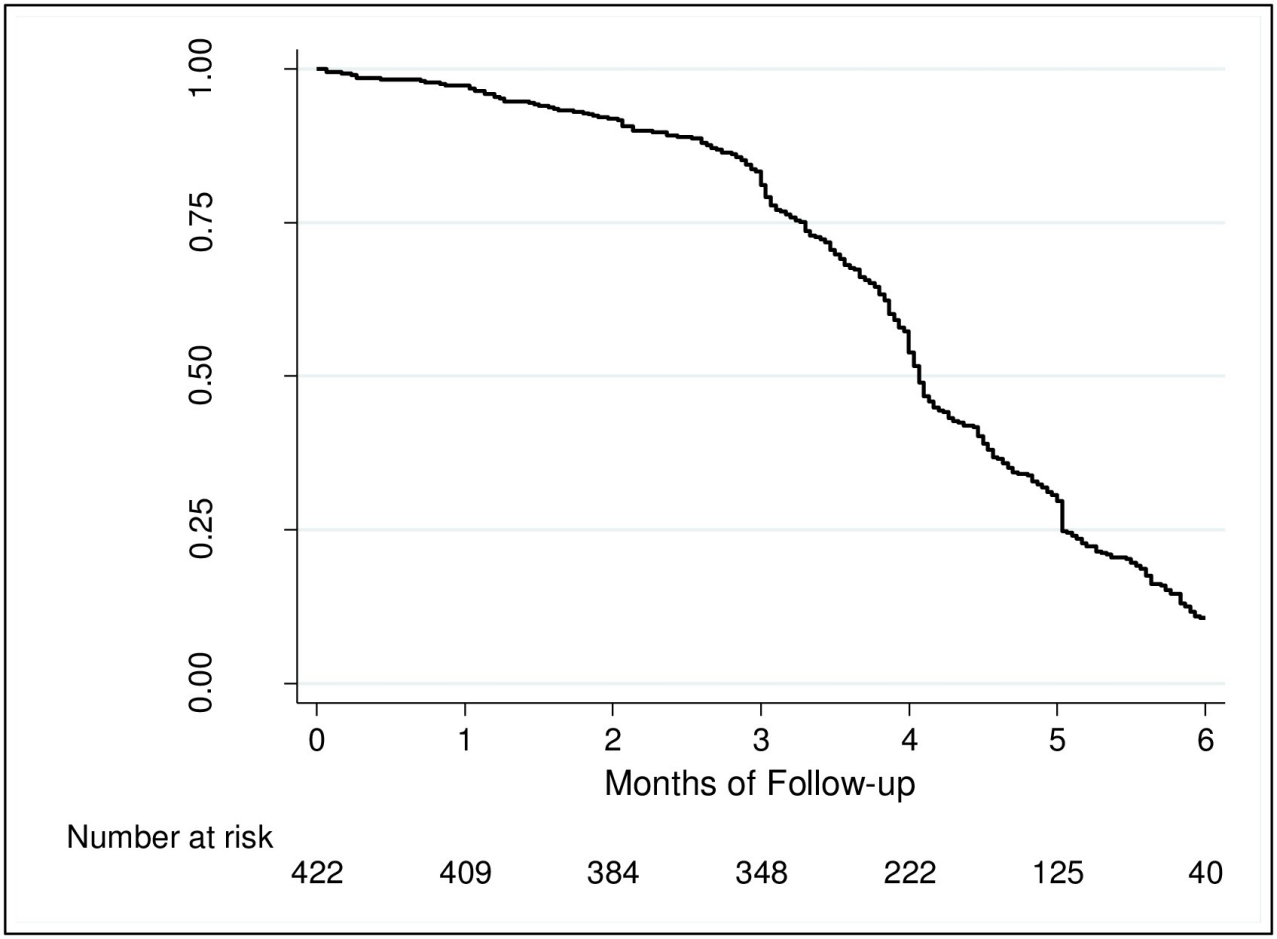
Cumulative probability of being predominantly breastfed.

**Fig 2:**
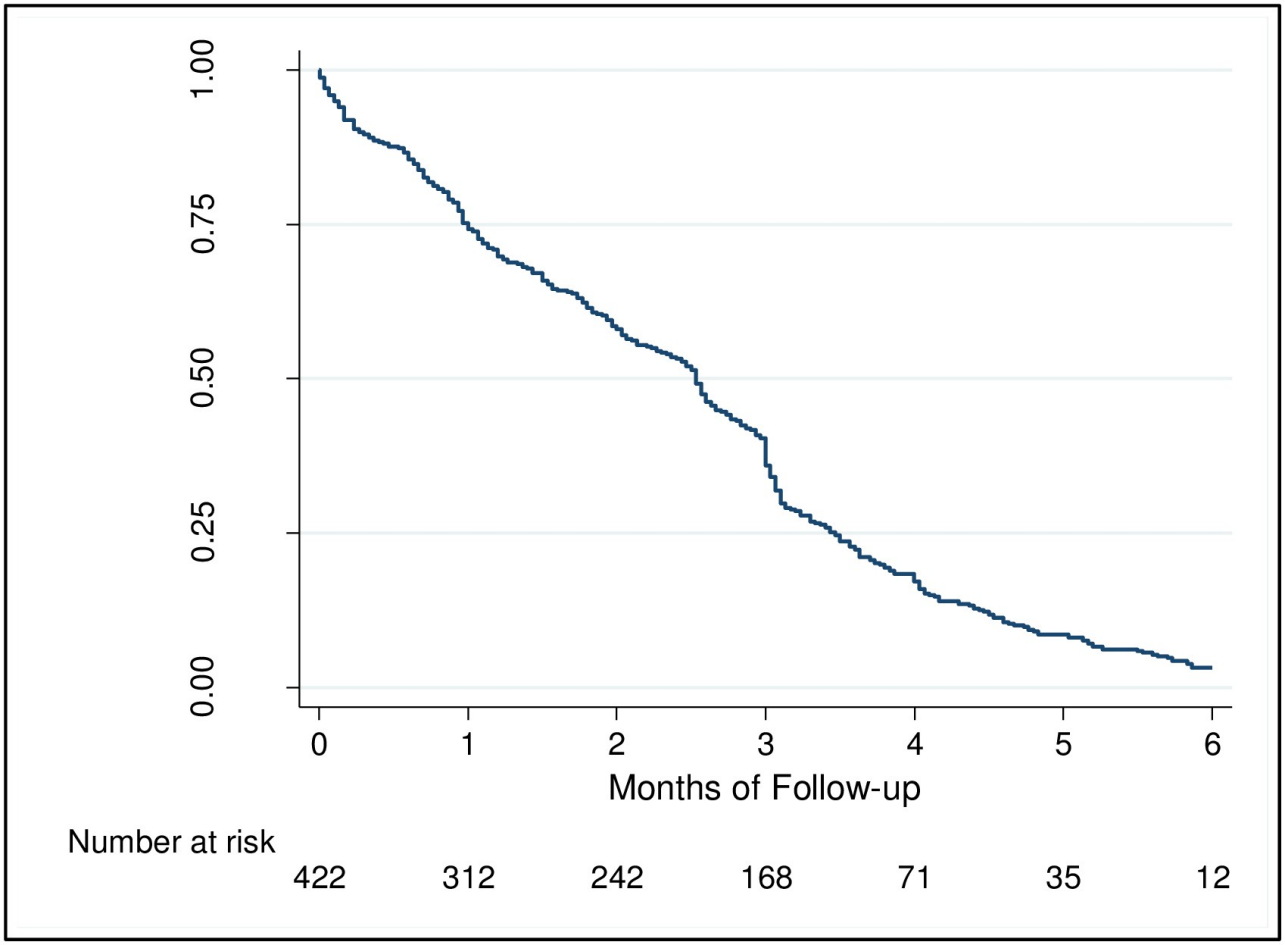
Cumulative probability of being exclusively breastfed.

All 422 mothers enrolled in the study initiated breastfeeding, 233 (55.2%) did it within an hour of birth. At six months, only 40 (9,5%) children were not yet fed with non-human milk or complementary foods (Fig 1), among then 12 (2.8%) were exclusively breastfed (Fig 2). The median duration of exclusive breastfeeding was 2.5 months (IQR 1.0 to 3.5) while the median duration of predominant breastfeeding was 4.1 months (IQR 3.3 to 5.0).

### 3.3. Frequency of breastfeeding problems

The frequency of breastfeeding problems during different periods, from the first week after delivery to the sixth month is presented in Table 2.

**Table 2:** Proportion of lactating mothers who encountered problems during different periods from delivery to six months.

Period	Frequency	Percentage [CI_95%_]
0.0–7.0 days	72/422	17.1 [13.7–21.1]
8.0–29 days	63/389	16.2 [12.8–20.3]
1.0–1.9 months	26/ 388	6.7 [4.5–9.8]
2.0–2.9 months	24/366	6.6 [4.3–9.7]
3,0–3,9 months	22/326	6.7 [4.4–10.2]
4,0–4,9 months	12/206	5.8 [3.0–10.0]
5,0–5,9 months	5/107	4.7 [1.5–10.6]
**Overall**	**140/422**	**33.2 [27.7**–**36.7]**

A lactating mothers could contribute for multiple periods

Overall, 140 of the 422 mothers included in the study experienced breastfeeding problems at least once during the first six months (32.2%; 95%CI 27.7–36.7). The problems were more common during the first month after delivery. Their frequency during the first week (17.1%; 95%CI 13.7–21.1) was similar to that of the rest of the first month (16.2%; 95%CI 12.8–20.3), and was three times higher than that of the following five months.

### 3.4. Types of breastfeeding problems

The types of breastfeeding problems experienced by lactating mothers are presented in Table 3.

**Table 3:** Type of breastfeeding difficulties encountered by mothers during different periods from delivery to six months.

Type of breastfeeding problem	0-7days	8–29 days	1,0–5,9 months
	n = 72	n = 63	n = 89
Cracked or wounded nipples	23(31.9)	23(36.5)	27(30.3)
Sore nipples during breastfeeding	22(30.6)	12(19.0)	9(10.1)
Insufficient production of milk	14(19.4)	10(15.9)	10(11.2)
Breast engorgement	5(6.9)	11(17.5)	12(13.5)
Pain in the operating wound	4(5.6)	2(3.2)	1(1.1)
Difficulty to take the right position	1(1.4)	3(4.8)	1(1.1)
Child’s sickness	0(0.0)	3(4.8)	21(23.6)
Mother’s sickness	0(0.0)	1(1.6)	4(4.5)
Breast abscess	0(0.0)	0(0.0)	4(4.5)

A mother could experience more than one breastfeeding problem

Cracked nipples were the most commonly reported breastfeeding problem. Almost one third of mothers reported this problem during the first week (31.9%) as well as during the rest of the first month (36.5%) and beyond the first month (30.3%).

Apart from cracked nipples, the other most common breastfeeding problems reported during the first month after delivery included sore nipples during breastfeeding, insufficient production of milk and breast engorgement: during the first week, the proportion of mothers who mentioned having those breastfeeding problems was respectively 30.6%, 19.4% and 6.9%; whereas during the rest of the first month, these proportions declined respectively to 19.0%, 15.9%, except for breast engorgement that increased to17.5%.

Beyond the first month, except for the cracked nipples already mentioned (30.2%), the most commonly reported breastfeeding problems included infant’s illness (23.6%), insufficient production of milk (11.2%) and sore nipples during breastfeeding (10.1%).

## 4. Discussion

This study showed that cracked nipples, sore nipples during breastfeeding, insufficient production of milk and breast engorgement were the breastfeeding problems most commonly encountered by lactating mothers. These breastfeeding problems occurred mainly during the first week after birth and throughout the rest of the first month.

During this study, the highest frequency of breastfeeding problems was noted within the first week after delivery (17.1%) and remained high during the rest of the first month (16.2%) but decreased from the second month (6.7%). At six months, less than one out of twenty breastfeeding mothers (4.7%) reported experiencing breastfeeding problems. This finding can be explained by the fact that in Kinshasa, a woman who has just given birth is regularly visited by relatives who provide her with breastfeeding technique support. Thus, over time, she acquires more and more experience in the practice of breastfeeding, which results in a decrease in the frequency of breastfeeding difficulties. These results reinforce the evidence that the first month after childbirth, and especially the first week, is critical for the establishment and the continuation of breastfeeding [18]. This observation suggests that the mother should be supported during this period to prevent and overcome breastfeeding difficulties. Higher proportions of breastfeeding problems (40–80%) have been reported during the first week in developed countries [19–21]. An explanation for this noticeable difference is the fact that breastfeeding is common in Africa; therefore, African women could be positive thinkers towards breastfeeding. Women who think positively about breastfeeding perceive problems as ‘normal’ while those who lack self-assurance in their capacity to breastfeed are more likely to focus on its negative aspects [22].

With regard to the type of breastfeeding problems encountered, the most reported were: cracked nipples; sore nipples during breastfeeding; insufficient production of breast milk; and breast engorgement. The same problems were also reported during the first month, with almost similar proportions in Denmark [20], the United States of America (USA) [21] and Ethiopia [23]. Cracked or sore nipples are mainly a consequence of poor breastfeeding technique [24] that can be prevented with good positioning, optimal attachment of the baby and gentle removal from the breast when the baby is satisfied [19, 25]. Therefore, breastfeeding technique should be assessed and taught to each mother at least once by midwives while at the maternity facility and corrected if needed [25].

Apart from cracked or sore nipples, insufficient production of breast milk was the second most common breastfeeding problem reported by mothers during this study. However, this insufficiency is more often secondary than primary [19]. The primary glandular insufficiency is rare. More often, insufficient milk production occurs: when the breasts are not emptied sufficiently and frequently for whatever reason, notably poor breastfeeding technique; painful latch on while suckling; mother and infant separation; and illness of mother or infant [25]. If the mother and infant are separated or unwell, early and regular milk expression by hand or pump should be started to maintain milk production [26]. On the other hand, insufficient production of breast milk is more perceived than real [22]. The mother’s perception of not having enough milk is often a misinterpretation of her crying baby or a feeling of soft or empty breasts. Therefore, the mother experiencing insufficient production of breast milk needs to be supported by midwives and relatives to improve her breastfeeding self-efficacy.

The third most common breastfeeding problem reported in this study was breast engorgement that, once again, result from a poor emptying of breasts [19]. Early initiation of breastfeeding, spending more time to breastfeed during the first 48 hours after childbirth and emptying one breast at each breastfeeding by alternating the breast that is first offered, may help to prevent this problem [25, 27].

The results of this study suggest that breastfeeding mothers benefit from breastfeeding support offered by midwives as soon as possible after the childbirth. While at the maternity facility, breastfeeding technique should be assessed and taught to each mother at least once. A training program on breastfeeding counselling for midwives should be developed for this purpose.

### 4.1. Strengths and limitations

The results of this study should be interpreted taking into account its strengths and limitations.

One of the limitations was that the data used were collected in 2013 and may seem outdated. However, in our opinion, results from this study are still valuable today, given that the national rate of mothers achieving the global recommendation on exclusive breastfeeding remained around 50% from 2013 to 2018 [7, 9]. This study had good external validity; it included a sufficient number of breastfeeding mothers selected using a completely random process. The study had sufficient power and less than 5% of lost to follow-up were noted.

Furthermore, this study was prospective; thus, the low reliability of the duration of exclusive breastfeeding determined retrospectively was avoided. However, the recall bias was not completely avoided because the day when foods or drinks other than breast milk were introduced was determined retrospectively. Therefore, mothers could or could not remember precisely that moment. Nonetheless, to our knowledge, this study is the first carried out in Kinshasa to investigate breastfeeding problems experienced by mothers.

### 4.2. Recommendation for future research

The most common breastfeeding problems reported during this study may be prevented by improving the breastfeeding technique. Furthermore, these problems occurred mainly during the first month after the childbirth, especially during the first week. Future research should be carried out to assess the effect of technical support, offered as early as possible after the childbirth to improve the breastfeeding technique, on the occurrence of these problems.

## 5. Conclusion

This study has demonstrated that lactating mothers experience several problems. The first four weeks after birth are the most critical to the occurrence of those problems. Most of these problems might be prevented with optimal breastfeeding technique and by emptying sufficiently and frequently the breasts. Therefore, breastfeeding mothers should be supported as soon as possible after delivery, to improve their breastfeeding technique and to be informed on how to maintain breast milk supply.

## Supporting information

S1 Appendix Data collection toll. (PDF)

S2 Appendix Code book. (PDF)

S1 Dataset Database. (SAV)

S1 Table Comparison of participants lost to follow-up with those completely followed. (PDF)

## Abbreviations

CI: Confidence interval
DRC: Democratic Republic of the Congo
IQR: Interquartile range
WHO: World Health Organization
