# Supplementary material for: Common breastfeeding problems experienced by lactating mothers during the first six months in Kinshasa
Source: PLoS One. 2022 Oct 12;17(10):e0275477. doi: 10.1371/journal.pone.0275477 (PMC9555666; doi:10.1371/journal.pone.0275477)
Supplement: S1 Appendix — (PDF) [file pone.0275477.s001.pdf]

Common breastfeeding problems experienced by lactating mothers during the first six months in Kinshasa

DATA COLLECTION TOOL

MODULE I: QUESTIONNAIRE FOR MOTHERS AT REGISTRATION

Section 1: IDENTIFICATION

Nº	Questions	Answers	Code
111	Maternity facility name		
112	Mother's first name		
113	Residency		
114	Telephone		

115. Date of interview /___/___/ /___/____/ /___/___/___/___/

N°	Investigation team	Names	Date	Signature
116	Interviewer			
117	Supervisor			
118	Codifier			
119	Entry clerk			

Start time : /___/___/ : /___/___/

Section 2: ELIGIBILITY

Instructions: Interviewer, complete this section using the mother's medical record.

N°	Question	Answer	Code
121	How old is the mother?<br>***If < 18 years, not eligible***	/___/___/ Years	
122	What was the age of pregnancy at the time of delivery?<br>***If < 37 weeks, not eligible***	/___/___/ (Weeks)	
123	How many children has she given birth?<br>***If >1, not eligible***	/___/	
124	Is the newborn alive?<br>***If not, not eligible***	1. Yes 2. No	
125	Did the newborn stay in an intensive care unit?<br>***If yes, not eligible***	1. Yes 2. No	
126	Does the mother have a morbid condition which made it impossible to initiate breastfeeding?<br>***If yes, not eligible***	1. Yes 2. No	

Section 3 : SOCIODEMOGRAPHIC DATA

N⁰	Question	Answer	Code
131	What is your level of education?	1. Has never been to school<br>2. Primary<br>3. Secondary<br>4. Academic	
132	What is your marital status?	1. Single<br>2. Married / Common-law<br>3. Divorced / Separated<br>4. Widow	
133	What is your main occupation?	1. Schoolgirl / student<br>2. Unemployed<br>3. Housewife<br>4. Trader<br>5. Farmer<br>6. Coiffeuse/ Fashion designer<br>7. Employed in the private sector<br>8. Employed in the public sector<br>9. Other (to be specified): ……………	
134	How many people are in your household?	/___/___/	
135	How much do you spend on average per day for food?<br>***(Include the cost of food taken from stock)***	1. /___/___/___/___/___/___/ CDF<br>2. Don't know	

Section 4: MOTHER AND CHILD BIOMEDICAL DATA

N°	Question	Answer	Code
141	How many times did you reached ANC visits during your current pregnancy?	/___/	
142	What date did you give birth?	/___ ___//___ ___//___ ___/<br>(date /month / year)	
143	By what route did you give birth?	1. Low track (Vaginal route)<br>2. High way (cesarean section)	

144	What is your child's gender?	1. Male<br>2. Female	
145	What was his/her birth weight?	/___/___/___/___/ (grams)	
146	Within the health facility where you followed the ANC visits and where you gave birth:		
	1. Have you been told about the benefits of breastfeeding?	1. Yes 2. No	
	2. Was your child put in skin-to-skin contact with you after birth?	1. Yes 2. No	
	3. After your baby was born, how long has it been before you breastfed?	1. Less than thirty minutes<br>2. Between 30 min and an hour<br>3. More than an hour	
	4. Were you helped to start breastfeeding within an hour after birth?	1. Yes 2. No	
	5. Has the staff made sure your baby was capable to suckle?	1. Yes 2. No	
	6. If necessary, did the staff help you to correct position and attachment of your child during breastfeeding?	1. Yes 2. No	
	7. Was a food/drink other than breast-milk given to your child?	1. Yes 2. No	
	8. Did you stay together with your child 24 hours a day?	1. Yes 2. No	
	9. Were you encouraged to breastfeed your baby on demand whether day or night?	1. Yes 2. No	
	10. Has a pacifier been given to your child?	1. Yes 2. No	
	Total:		

Section 5 : CONDUCTING BREASTFEEDING

N°	Question	Answer	Code
151	How old is now your baby?	/___/___/ days	
152	What's his/her name?	……………………………………..	
153	What food/drink did [Baby's Name] received yesterday?<br><br>***Many answers are possible. If the answer is only 1, go to question 155***	1. Breast-milk<br>2. Breast-milk substitutes<br>3. Water/fruit juice/ritual product<br>4. Porridge<br>5. Family dish<br>6. Other (to be specified: ………	
154	How long have you been giving the food/drink ………………………. to [Baby's Name]?		
	2. Breast-milk substitutes	/___/___/ days	
	3. Water/fruit juice/ritual product	/___/___/ days	
	4. Porridge	/___/___/ days	
	5. Family dish	/___/___/ days	
	6. Other (to be specified: ……………..	/___/___/ days	
155	Have you experienced any breastfeeding difficulty?<br>***If the answer is 2, go to question 157***	1. Yes<br>2. No	
156	If yes, which one (s)?		
157	What was the first meal received by [Baby's Name] after birth?	1. Breast-milk<br>2. Water (Flat or Sweet)<br>3. Breast-milk substitutes<br>4. Ritual product<br>5. Other (to be specified): ………	
158	Did you give [Baby's Name] the first yellowish milk that came out of your breasts?<br>***If yes, go to question 1510***	1. Yes<br>2. No	
159	If no, why didn't you give it?	1. Nothing/Don't know<br>2. This milk was dirty<br>3. Other (to be specified): ……….	

1510	Did you give [Baby Name] anything else to drink while waiting for the milky rise?<br>***If no, end of interview***	1. Yes<br>2. No				
1511	If yes, which one?<br><br>***Many answers are possible***	1. Sweet water<br>2. Breast-milk substitutes<br>3. Ritual product<br>4. Other (to be specified): ………				
1512	Why did you give him/her this drink?	1. Nothing<br>2. Baby was hungry/crying<br>3. Out of respect for our custom<br>4. Other (to be specified): ……				
		1	2	3	4	
	1. Sweet water					
	2. Breast-milk Substitutes					
	3. Ritual product					
	4. Other (to be specified): …………….					

Thanks for your collaboration.

End time: /___/___/: /___/___/

MODULE II : QUESTIONNAIRE FOR MOTHERS DURING MONTHLY FOLLOW-UP VISITS

Section 1: IDENTIFICATION

Nº	**Questions**	**Answers**	**Code**
211	Mothers' first name		
212	Residency		
213	Telephone		

216. Date of interview /___/___/   /___/____/ /___/___/___/___/

**N°**	**Investigation team**	**Names**	**Date**	**Signature**
217	Interviewer			
218	Supervisor			
219	Codifier			
210	Entry clerk			

Start time : /___/___/ : /___/___/

Section 2: BREASTFEEDING DIFFICULTIES

**N°**	**Question**	**Answers**	**Code**
221	Since the last interview, have you encountered any difficulties with breastfeeding?<br>***If no, go to question 3123***	1. Yes<br>2. No	
222	If yes, which one (s)?		
223	What was the effect of …………………… on breastfeeding?<br>***(Mention the first difficulty cited)***	1. No effect<br>2. Small difficulty<br>3. Great difficulty	

Section 3: CURRENT INFANT'S FEEDING

N°	Question	Answer					Code
231	What food/drink did your child's diet consisted of yesterday?<br>***Many answers are possible. If the answer is only 1, end the interview***	1. Breast-milk<br>2. Breast-milk substitutes<br>3. Water/fruit juice/ritual product<br>4. Porridge<br>5. Family dish<br>6. Other (to be specified: ………					
232	How long have you been giving your child?						
	2. Breast-milk substitutes	/___/___/ days					
	3. Water/fruit juice/ritual product	/___/___/ days					
	4. Porridge	/___/___/ days					
	5. Family dish	/___/___/ days					
	6. Other (to be specified: ……………………	/___/___/ days					
233	Why, in addition to breast-milk, did you give …….to your child?<br><br>***List all other foods/drinks given to the child.***	1. I didn't have enough milk<br>2. The child was not satiated<br>3. It was very hot<br>4. The child cried a lot<br>5. Other (to be specified): ………					
	2. Breast-milk substitutes	1	2	3	4	5	
	3. Water/fruit juice/ritual product						
	4. Porridge						
	5. Family dish						
	6. Other (to be specified: ……………………						

Thanks for your collaboration.

End time: /___/___/: /___/___/
