# Supplementary material for: Common breastfeeding problems experienced by lactating mothers during the first six months in Kinshasa
Source: PLoS One. 2022 Oct 12;17(10):e0275477. doi: 10.1371/journal.pone.0275477 (PMC9555666; doi:10.1371/journal.pone.0275477)
Supplement: S2 Appendix — (PDF) [file pone.0275477.s002.pdf]

Common breastfeeding problems experienced by lactating mothers during the first six months in Kinshasa

CODE BOOK

Variable	Label	Type	Code
NUMFICHE	ID number	Interval	
AGE	Age	Interval	Years
AGECAT	Categorized age	Nominal	0= "≥30 years"<br>1= "20-29 years"<br>2= "<20 years"
INSTRUC	Educational level	Ordinal	1= Never been at school<br>2= Primary<br>3= Secondary<br>4= University
ETCIVILCAT	Categorized marital status	Nominal	0=Living with a partner<br>1=Single
OCCUPCAT	Categorized occupation	Nominal	1= Student<br>2= Housewife<br>3= Small trade<br>4= Farm worker<br>5= Hairdresser/dressmaker<br>6= Paid job
PARITE	Parity	Interval	
PARITECAT	Categorized parity	Nominal	0= "≥4"<br>1= "2-3"<br>3= "1"
DIFFI0	Difficulties during the first week?	Nominal	0=No<br>1=Yes
DIFFIOA	Type of difficulty during the first week A	Nominal	1= Insufficient production of milk<br>2= Inverted nipple<br>3= Cracked or wounded nipples<br>4= Breast engorgement<br>5= Sore nipples during breast-feedings<br>6= Difficulty to take the right position<br>7= Pain in the operating wound<br>8= Ulcers in the child's mouth
DIFFIOB	Type of difficulty during the first week B	Nominal	The same categories as DIFFIOA
DIFFIOC	Type of difficulty during the first week C	Nominal	The same categories as DIFFIOA
DIFFIOD	Type of difficulty during the first week D	Nominal	The same categories as DIFFIOA
VISITE1	First follow-up visit	Nominal	1=No<br>2=Yes
DIFFI1	Difficulties during the first month?	Nominal	0=No<br>1=Yes
DIFFI1A	Type of difficulty during the first month A	Nominal	1= Insufficient production of milk<br>2= Cracked or wounded nipples<br>3= Sore nipples during breast-feedings<br>5= Child's sickness<br>6= Breast engorgement<br>7= Ulcers in the child's mouth<br>8= Pain in the operating wound<br>9= Difficulty to take the right position<br>10. Mother's sickness

			11. Inverted nipple<br>12. Breast abscess<br>13. Baby bites the nipple during breast feeds<br>14. Baby's brother's illness<br>15. I have too much work to do
DIFFI1B	Type of difficulty during the first month B	Nominal	The same categories as DIFFI1A
DIFFI1C	Type of difficulty during the first month C	Nominal	The same categories as DIFFI1A
DIFFI1D	Type of difficulty during the first month D	Nominal	The same categories as DIFFI1A
VISITE2	Second follow-up visit	Nominal	1=No<br>2=Yes
DIFFI2	Difficulties during the second month?	Nominal	0=No<br>1=Yes
DIFFI2A	Type of difficulty during the second month A	Nominal	The same categories as DIFFI1A
DIFFI2B	Type of difficulty during the second month B	Nominal	The same categories as DIFFI1A
DIFFI2C	Type of difficulty during the second month C	Nominal	The same categories as DIFFI1A
DIFFI2D	Type of difficulty during the second month D	Nominal	The same categories as DIFFI1A
VISITE3	Third follow-up visit	Nominal	1=No<br>2=Yes
DIFFI3	Difficulties during the third month?	Nominal	0=No<br>1=Yes
DIFFI3A	Type of difficulty during the third month A	Nominal	The same categories as DIFFI1A
DIFFI3B	Type of difficulty during the third month B	Nominal	The same categories as DIFFI1A
DIFFI3C	Type of difficulty during the third month C	Nominal	The same categories as DIFFI1A
DIFFI3D	Type of difficulty during the third month D	Nominal	The same categories as DIFFI1A
VISITE4	Fourth follow-up visit	Nominal	1=No<br>2=Yes
DIFFI4	Difficulties during the fourth month?	Nominal	0=No<br>1=Yes
DIFFI4A	Type of difficulty during the fourth month A	Nominal	The same categories as DIFFI1A
DIFFI4B	Type of difficulty during the fourth month B	Nominal	The same categories as DIFFI1A
DIFFI4C	Type of difficulty during the fourth month C	Nominal	The same categories as DIFFI1A
DIFFI4D	Type of difficulty during the fourth month D	Nominal	The same categories as DIFFI1A
VISITE5	Fifth follow-up visit	Nominal	1=No<br>2=Yes
DIFFI5	Difficulties during the fifth month?	Nominal	0=No<br>1=Yes
DIFFI5A	Type of difficulty during the fifth month A	Nominal	The same categories as DIFFI1A
DIFFI5B	Type of difficulty during the fifth month B	Nominal	The same categories as DIFFI1A
DIFFI5C	Type of difficulty during the fifth month C	Nominal	The same categories as DIFFI1A
DIFFI5D	Type of difficulty during the fifth month D	Nominal	The same categories as DIFFI1A
VISITE6	Sixth follow-up visit	Nominal	1=No<br>2=Yes
DIFFI6	Difficulties during the sixth month?	Nominal	0=No<br>1=Yes
DIFFI6A	Type of difficulty during the sixth month A	Nominal	The same categories as DIFFI1A
DIFFI6B	Type of difficulty during the sixth month B	Nominal	The same categories as DIFFI1A
DIFFI6C	Type of difficulty during the sixth month C	Nominal	The same categories as DIFFI1A
DIFFI6D	Type of difficulty during the sixth month D	Nominal	The same categories as DIFFI1A

AGEAPREJ	Duration of exclusive or predominant breastfeeding in days	Interval	Days
AGEAPRES	Duration of exclusive or predominant breastfeeding in days	Interval	Weeks
APRE01	Cessation of exclusive or predominant breastfeeding during the first month	Nominal	0=No<br>1=Yes
APRE02	Cessation of exclusive or predominant breastfeeding during the second month	Nominal	0=No<br>1=Yes
APRE03	Cessation of exclusive or predominant breastfeeding during the third month	Nominal	0=No<br>1=Yes
APRE04	Cessation of exclusive or predominant breastfeeding during the fourth month	Nominal	0=No<br>1=Yes
APRE05	Cessation of exclusive or predominant breastfeeding during the fifth month	Nominal	0=No<br>1=Yes
APRE06	Cessation of exclusive or predominant breastfeeding during the sixth month	Nominal	0=No<br>1=Yes
