# Supplementary material for: Common breastfeeding problems experienced by lactating mothers during the first six months in Kinshasa
Source: PLoS One. 2022 Oct 12;17(10):e0275477. doi: 10.1371/journal.pone.0275477 (PMC9555666; doi:10.1371/journal.pone.0275477)
Supplement: S1 Table — (PDF) [file pone.0275477.s004.pdf]

Common breastfeeding problems experienced by lactating mothers during the first six months in Kinshasa

SUPPLEMENTAL TABLE

Table 1. Comparison of participants lost to follow-up with those completely followed

	**All**<br><br>n=422	**Completely followed**<br>n(%)<br>405(96.0)	**Lost to follow up**<br>n(%)<br>17(4.0)	**p-value***
**Age (years)**				
<20	47	43(91.5)	4(8.5)	0.106
20-29	241	235(97.5)	6(2.5)	
≥30	133	126(94.7)	7(5.3)	
**Education level**				
Never been at school	8	8 (100.0)	0(0.0)	0.604
Primary	207	196(94.7)	11(5.3)	
Secondary	179	173(96.6)	6(3.4)	
University	28	28(100.0)	0(0.0)	
**Marital status**				
Living with a partner	360	348(96.7)	12(3.3)	0.088
Single	62	57(91.9)	5(8.1)	
**Occupation**				
Housewife	204	197(96.6)	7(3.4)	0.362
Worker/Student	218	208(95.4)	10(4.6)	
**Parity**				
1	162	155(95.7)	7(4.3)	0.627
2-3	167	162(97.0)	5(3.0)	
≥4	93	88(94.6)	5(5.4)	

*Pearson's Chi-square test or, if not applicable, Fisher's exact test

Table 2: Association between problems encountered during a given period and the non-continuation of exclusive breastfeeding

Period (months)	Breastfeeding problems		Events	Persons-weeks	HR* ($CI_{95\%}$)	p-value
0.0 - 0.9	yes	105	44	314.14	2.72(1.84-4.01)	<0.001
	No	317	60	1168.86	1	
1.0 - 1.9	yes	26	9	177.14	2.64(1.30-5.35)	0.007
	No	362	54	2805.86	1	
2.0 - 2.9	yes	24	6	273.11	1.29(0.56-2.99)	0.545
	No	342	68	4025.86	1	
3.0 - 3.9	yes	22	8	281.57	1.67(0.81-3.46)	0.167
	No	304	80	4710.71	1	
4.0 - 4.9	yes	12	6	102.69	13.40(2.67-67.24)	0.002
	No	194	32	7341.43	1	
5.0 - 5.9	yes	5	0	120.00	0.36 (0.13-12.21)	0.652
	No	102	20	8728.25		

* Hazard ratio
